# ALIGN-CARE: IMPLEMENTATION AND FEASIBILITY OF NEW GERIATRIC SURGERY CO-MANAGEMENT MODEL FOR PHYSICAL AND SOCIAL FRAILTY

**DOI:** 10.1093/geroni/igac059.2116

**Published:** 2022-12-20

**Authors:** Fred Ko, Stephanie Chow, Abigail Baim-Lance, Amy Park, Gabrielle Schiller, Minji Kim, Kavya Sreevalsan, William Hung

**Affiliations:** Icahn School of Medicine at Mount Sinai, New York, New York, United States; Icahn School of Medicine at Mount Sinai, New York, New York, United States; Icahn School of Medicine at Mount Sinai, New York, New York, United States; Icahn School of Medicine at Mount Sinai, New York, New York, United States; CUNY School of Public Health, Astoria, New York, United States; Icahn School of Medicine at Mount Sinai, New York, New York, United States; Icahn School of Medicine at Mount Sinai, New York, New York, United States; Icahn School of Medicine at Mount Sinai, New York, New York, United States

## Abstract

Pre-operative geriatric evaluation in frail older patients that addresses both medical and psychosocial needs is often not completed prior to surgery, leaving frail older adults ill prepared for surgery. The ALIGN-CARE interprofessional co-management model uniquely serves to standardize care and coordination amongst the surgeon, geriatrician and social worker in ambulatory settings to allow a greater window of time to intervene for surgical optimization. Program elements include assessment of preoperative medications, functional status, cognition, nutrition, What Matters Most, advance care planning (ACP), and social determinants of health. Recommendations and action items from the geriatric-social work team are presented to surgeon and primary care physicians for follow-up and optimization. Program and process elements recorded in a REDCap database facilitate longitudinal evaluation of ALIGN-CARE model implementation feasibility. Initial data of 9 frail surgical oncology patients indicate increases in meaningful ACP discussions, patient-oriented decisions regarding surgery candidacy, and surgeon referrals for inpatient geriatric consultation post-operatively as a result of ALIGN-CARE. Current analyses to assess feasibility and quality of the program include thematic coding of program documents, semi-structured interviews of key stakeholders (surgeons, geriatricians, social workers, patients), and quantitative analysis of program outcomes, including uptake of program elements and process measures. Planned areas of analytic focus include workflow, cross-specialty coordination, and patient and primary care engagement and activation. We anticipate identifying specific barriers and facilitators of ALIGN-CARE implementation in our evaluation to aid the dissemination and upscaling of interprofessional co-management programs aimed to improve outcomes and quality of life for frail, older surgical patients.

